# Systemic Inflammation Evaluated by Interleukin-6 or C-Reactive Protein in Critically Ill Patients: Results From the FROG-ICU Study

**DOI:** 10.3389/fimmu.2022.868348

**Published:** 2022-05-12

**Authors:** Adrien Picod, Louis Morisson, Charles de Roquetaillade, Malha Sadoune, Alexandre Mebazaa, Etienne Gayat, Beth A. Davison, Gad Cotter, Benjamin Glenn Chousterman

**Affiliations:** ^1^ Department of Anesthesiology, Burn and Critical Care, University Hospitals Saint-Louis—Lariboisière, AP-HP, Paris, France; ^2^ UMR-S 942, Institut National de la Santé et de la Recherche Médicale (INSERM), Cardiovascular Markers in Stressed Conditions (MASCOT), Paris University, Paris, France; ^3^ Sorbonne University, Paris, France; ^4^ Department of Anesthesiology and Pain Medicine, Hôpital Maisonneuve-Rosemont, CIUSSS de l’Est de l’Ile de Montréal, Montréal, QC, Canada; ^5^ Momentum Research Inc., Durham, NC, United States

**Keywords:** interleukin-6, Simplified Acute Physiology Score, critical illness, biomarkers, C-reactive protein, Sequential organ failure assessment, sepsis

## Abstract

**Background:**

The prognostic impact of high concentration of interleukin-6 (IL-6) or C-reactive protein (CRP), two routinely available markers of systemic inflammation in the general population of critically ill patients, remains unclear. In a large cohort of critically ill patients including septic and non-septic patients, we assessed the relationship between baseline IL-6 or CRP and mortality, organ dysfunction, and the need for organ support.

**Methods:**

This was an ancillary analysis of the prospective French and euRopean Outcome reGistry in Intensive Care Units (FROG-ICU) study including patients with a requirement for invasive mechanical ventilation and/or vasoactive drug support for more than 24 h following intensive care unit (ICU) admission. The primary objective was to determine the association between baseline IL-6 or CRP concentration and survival until day 90. Secondary outcomes included organ dysfunction as evaluated by the Sequential Organ Failure Assessment (SOFA) score, and the need for organ support, including vasopressors/inotropes and/or renal replacement therapy (RRT).

**Results:**

Median IL-6 and CRP concentrations (*n* = 2,076) at baseline were 100.9 pg/ml (IQR 43.5–261.7) and 143.7 mg/L (IQR 78.6–219.8), respectively. Day-90 mortality was 30%. High IL-6 or CRP was associated with worse 90-day survival (hazard ratios 1.92 [1.63–2.26] and 1.21 [1.03–1.41], respectively), after adjustment on the Simplified Acute Physiology Score II (SAPS-II). High IL-6 was also associated with the need for organ-support therapies, such as vasopressors/inotropes (OR 2.67 [2.15–3.31]) and RRT (OR 1.55 [1.26–1.91]), including when considering only patients independent from those supports at the time of IL-6 measurement. Associations between high CRP and organ support were inconsistent.

**Conclusion:**

IL-6 appears to be preferred over CRP to evaluate critically ill patients’ prognoses.

## Introduction

Interleukin-6 (IL-6) is a pleiotropic 26-kDa cytokine involved in numerous signaling pathways during both homeostasis and disease. Notably, IL-6 is implicated in the initiation and regulation of the host inflammatory response to septic and non-septic aggressions. During disease, IL-6 mediates fever, loss of appetite, weight loss, and anemia, and constitutes the main inductor of acute-phase proteins synthesis, including C-reactive protein (CRP) (1). Additionally, and relevant to the specific context of critical illness, IL-6 has been implicated in the development of vascular hyperpermeability, myocardial depression, and activation of coagulation, and correlates with vasoplegia during septic shock (2–5). Plasma concentration of IL-6 is subject to wide variations, ranging from 0 to 7 pg/ml in healthy subjects up to concentrations higher than 1 μg/ml during septic shock (6). Given its key role in the genesis of pro-inflammatory processes, it is commonly accepted, especially in the field of autoimmune diseases and cancer, that plasma IL-6 concentration represents a better predictor of the activity of the disease than other inflammatory markers such as CRP (1).

The negative prognostic impact of high IL-6 concentration has been recently highlighted during the SARS-CoV-2 pandemic, as an independent predictor of the severity of and mortality due to the coronavirus disease 2019 (7). Previously, numerous studies have examined the prognostic role of IL-6 concentration on intensive care unit (ICU) admission during sepsis, almost constantly finding a negative impact of high concentrations on survival (8). However, very little research has focused on other critically ill populations. These works, mainly small-sized and focused on acute heart failure, nevertheless seem to find a similar prognostic impact. Interestingly, high IL-6 concentrations in these non-septic patients appeared to be associated with the onset of vasoplegic shock, systemic hypoperfusion, and multiple organ failure (9–11). Conversely, the prognostic value of CRP, although routinely used as a marker of systemic inflammation, has been poorly investigated so far. A synthesis of the work carried out finds no prognostic interest of early CRP measurement in the ICU (12). However, these studies are heterogenous and most are limited to septic patients. Additionally, side-by-side comparison of CRP and IL-6 for prognostication has seldom been performed, although such analysis might have practical implications now that fast and relatively inexpensive IL-6 assays are broadly available.

Thus, it remains unclear whether high IL-6 concentration is associated with worse prognosis in the general population of critically ill patients, admitted for a wide range of septic and non-septic diagnoses and whether IL-6 yields a superior value than CRP in assessing prognosis.

In the present study, we aimed at assessing the association between IL-6 or CRP concentration and survival, the development of organ dysfunction, and the need for organ support among patients included in a large multicentric cohort of ICU patients.

## Materials and Methods

### Study Design and Population

This study is an ancillary analysis of the prospective multicenter observational cohort of the French and euRopean Outcome reGistry in Intensive Care Units (FROG-ICU) study (13). Details about design and data collection have been reported previously (14). Briefly, FROG-ICU study aimed at understanding long-term outcomes after ICU discharge as well as risk factors for morbidity and mortality. All consecutive patients from 21 medical, surgical, or mixed ICUs in France and Belgium between August 2011 and June 2013 were included if they required invasive mechanical ventilation and/or vasoactive drug support for more than 24 h after ICU admission. Patients were followed until death or up to 1 year after ICU discharge. Clinical and biological data were recorded at admission and during ICU stay including severity scores such as Simplified Acute Physiology Score II (SAPS-II) and Sequential Organ Failure Assessment (SOFA) as well as the use of life-sustaining therapies such as vasopressors/inotropes, renal replacement therapy (RRT), or invasive mechanical ventilation. The study received ethical committee approvals (Comité de Protection des Personnes—Ile de France IV, IRB n°00003835 and Commission d’éthique biomédicale hospitalo-facultaire de l’hôpital de Louvain, IRB n°B403201213352).

The primary objective of the present study was to assess the prognostic impact of plasma IL-6 and CRP concentrations at inclusion in the FROG-ICU study by assessing their association with 90-day survival. Secondary objectives focused on the relationships between these two biomarkers and organ dysfunction, life-sustaining therapies, as well as added prognostic value of IL-6 or CRP on top of SAPS-II or SOFA score.

### Sample Collection and Measurements

As part of the FROG-ICU study protocol, each patient had plasma samples collected at inclusion. These prospectively collected samples were used to determine baseline IL-6 (Elecsys COBAS—Roche Diagnostics, Penzberg, Switzerland) and CRP (Architect c—Abbott, Chicago, USA) concentrations in a central laboratory.

### Statistical Analyses

Results were expressed as median [interquartile range (IQR)] or number (percentage) as appropriate. In the absence of a predefined cutoff for the two biomarkers in a population of critically ill patients, data were described after dichotomization according to the median value of each biomarker. Normal distribution of the biomarkers was evaluated and, if appropriate, log_10_ transformation was performed. Differences between groups were assessed using the Mann–Whitney *U*-test or the Fischer exact test as appropriate. Rank correlation between IL-6 or CRP and other variables was assessed using Spearman’s rank correlation coefficient (*r*_s_). Survival curves plotted by the Kaplan–Meier method using median values of IL-6 and CRP were used for illustrative purposes, and differences between groups were tested with the log-rank test. Association between biomarkers and survival after adjustment for SAPS-II were estimated using a Cox proportional hazard model with results presented as adjusted hazard ratios (aHRs) and 95% confidence intervals (CIs). Subgroup analyses were performed for septic/non-septic patients, severity (below or above the median SAPS-II), age (below or above the median age), and gender (female/male). In order to better describe the relationship between biomarkers and mortality, continuous association between CRP or IL-6 and day-90 mortality was modelized using restricted cubic splines. Logistic regression was used to estimate the odds ratio (OR) of day-90 mortality per incremental log_10_ IL-6 as well as per 100 mg/L increment of CRP. Associations between biomarkers and organ support therapies were estimated using logistic regression with results presented as OR and 95% CI.

To explore the ability of these biomarkers to improve mortality prediction in comparison with classically used severity scores, category-free reclassification analyses were performed and net reclassification indexes (NRI) as well as integrative discrimination improvements (IDI) were calculated for each biomarker alone as well as in combination using previously described methodology (15). SAPS-II and SOFA scores were used as the reference predictor of mortality (16, 17). Areas under the receiver operating characteristic curves of the severity scores with or without addition of biomarkers values were compared using the DeLong test.

All statistical analyses were performed using the R statistical software version 4.0.2 (The R Foundation for Statistical Computing, Vienna, Austria) with the ggplot2, survminer, survival, Hmisc, dplyr, pROC, and forestplot packages. Each analysis was performed on available data with no imputation for missing values. A two-sided *p*-value of 0.05 was considered statistically significant.

## Results

### Study Population

Between August 2011 and June 2013, 2,087 patients were included in the FROG-ICU study, of whom 2,076 had both plasma IL-6 and CRP concentrations measured at baseline. Patient characteristics including demographic data, comorbidities, severity scores, admission diagnosis category, physiological and biological data, use of organ support, and outcomes are presented in **Table 1**. The main admission diagnoses were as follows: sepsis and septic shock (25.7%), acute respiratory failure (18.9%), and cardiac diseases, including cardiac arrest and cardiogenic shock (15.5%). Overall mortality at day 28 and day 90 were 22% and 30%, respectively.

**Table 1:** Patients’ characteristics according to median IL-6 or CRP.

	**Available *N* (%)**	**All patients *N* = 2076**	**IL-6^LOW^ *N* = 1038**	**IL-6^HIGH^ *N* = 1038**	***p***	**CRP^LOW^ *N* = 1038**	**CRP^HIGH^ *N* = 1038**	***p***
**Patients’ characteristics**								
Age, years [IQR]	2076 (100)	63 [51, 74]	63 [50, 74]	63 [51, 75]	0.584	62 [49, 73]	64 [52, 75]	0.013
Gender, female *n* (%)	2076 (100)	723 (34, 8)	402 (38.7)	321 (30.9)	<0.001	390 (37.6)	333 (32.1)	0.010
**SAPS-II, points [IQR]**	2075 (99.9)	49 [36, 63]	48 [35, 62]	49 [36, 63]	0.175	50 [36, 63]	48 [35, 62]	0.434
**SOFA, points [IQR]**	1510 (72.7)	8 [5, 10]	7 [4, 10]	8 [5, 11]	0.002	8.00 [5, 10]	8.00 [5, 10]	0.717
**Comorbidities**								
Charlson comorbidity index	2076 (100)	1 [0, 3]	1 [0, 2]	1 [0, 3]	0.044	1 [0, 3]	1 [0, 2]	0.220
Hypertension, *n* (%)	2072 (99.8)	898 (43.3)	441 (42.6)	457 (44.1)	0.506	416 (40.2)	482 (46.5)	0.004
Diabetes mellitus, *n* (%)	2072 (99.8)	383 (18.5)	210 (20.3)	173 (16.7)	0.042	185 (17.9)	198 (19.1)	0.497
Chronic heart failure, *n* (%)	2072 (99.8)	152 (7.3)	75 (7.2)	77 (7.4)	0.933	79 (7.6)	73 (7.0)	0.674
Chronic kidney disease, *n* (%)	2072 (99.8)	240 (11.6)	103 (9.9)	137 (13.2)	0.023	105 (10.1)	135 (13.0)	0.046
Chronic liver disease, *n* (%)	2072 (99.8)	158 (7.6)	65 (6.3)	93 (9.0)	0.025	120 (11.6)	38 (3.7)	<0.001
Cancer, *n* (%)	2072 (99.8)	279 (13.5)	107 (10.3)	172 (16.6)	<0.001	122 (11.8)	157 (15.2)	0.029
**Admission category**	2076 (100)							
Sepsis and septic shock*, *n* (%)		533 (25.7)	222 (21.4)	311 (30.0)	<0.001	209 (20.1)	324 (31.2)	<0.001
Hemorrhagic shock, *n* (%)		110 (5.3)	36 (3.5)	74 (7.1)	<0.001	61 (5.9)	49 (4.7)	0.281
Cardiac arrest or cardiogenic shock, *n* (%)		322 (15.5)	176 (17.0)	146 (14.1)	0.079	188 (18,1)	134 (12.9)	0.001
Renal and metabolic disease, *n* (%)		33 (1.6)	18 (1.7)	15 (1.4)	0.726	20 (1.9)	13 (1.3)	0.292
Neurological disorder, *n* (%)		284 (13.7)	193 (18.6)	91 (8.8)	<0.001	186 (17.9)	98 (9.4)	<0.001
Acute respiratory failure, *n* (%)		392 (18.9)	239 (23.0)	153 (14.7)	<0.001	217 (20.9)	175 (16.9)	0.021
Planned surgery, *n* (%)		165 (7.9)	55 (5.3)	110 (10.6)	<0.001	61 (5.9)	104 (10.0)	0.001
Trauma, *n* (%)		89 (4.3)	27 (2.6)	62 (6.0)	<0.001	24 (2.3)	65 (6.3)	<0.001
Others, *n* (%)		148 (7.1)	72 (6.9)	76 (7.3)	0.798	72 (6.9)	76 (7.3)	0.798
**Clinical and biological data at admission**								
GCS, points [IQR]	1301(62.7)	12 [3, 15]	13 [3, 15]	10 [3, 15]	0.002	12 [3, 15]	11 [3, 15]	0.490
PaO_2_/FiO_2_, mmHg [IQR]	1776 (85.5)	250 [174, 340]	263 [192, 357]	234 [160, 317]	<0.001	263 [193, 357]	235 [162, 313]	<0.001
Heart rate, beats/min [IQR]	1999 (96.3)	91 [78, 106]	88 [75, 103]	95 [82, 110]	<0.001	88 [75, 103]	95 [81, 109]	<0.001
SAP, mmHg [IQR]	2027 (97.6)	122 [108, 139]	127 [111, 144]	117.00 [105, 134]	<0.001	125 [110, 143]	120 [107, 136]	<0.001
DAP, mmHg [IQR]	1957 (94.3)	61 [53, 70]	64 [55, 73]	58 [52, 67]	<0.001	63 [54, 72]	60 [52, 68]	<0.001
WBC, 10^9^/L [IQR]	1933 (93.1)	10.9 [7.6, 16.2]	10.8 [7.9, 15.8]	11.2 [7.4, 16.6]	0.685	10.3 [7.4, 15.4]	11.5 [7.9, 16.9]	0.002
Urine output, ml [IQR]	1711 (82.4)	1350 [800, 2200]	1450 [900, 2300]	1253 [688, 1950]	<0.001	1400 [834, 2300]	1300 [733, 2000]	0.001
Creatinine, µmol/L [IQR]	1987 (95.7)	84 [59, 151]	76 [56, 129]	99 [64, 169]	<0.001	77 [56, 134]	94 [64, 165]	<0.001
Lactate, µmol/L [IQR]	1638 (78.9)	1.4 [1.0, 2.0]	1.3 [0.9, 1.7]	1.50 [1.1, 2.2]	<0.001	1.4 [1.0, 1.9]	1.5 [1.1, 2.0]	0.004
Bilirubin, µmol/L [IQR]	1293 (62.3)	13 [8, 27]	11 [7, 20]	15 [9, 34]	<0.001	12 [8, 29]	13 [8, 26]	0.536
PT rate, % [IQR]	1147 (55.3)	69 [54, 80]	73 [62, 84]	64 [50, 75]	<0.001	70 [53, 81]	68 [55, 79]	0.203
Platelets, 10^9^/L [IQR]	1950 (93.9)	164 [99, 243]	188 [119, 265]	139 [86, 220]	<0.001	169 [106, 252]	159 [94, 236]	0.038
**Organ support at admission**								
Vasopressors, *n* (%)	2069 (99.7)	1484 (71.7)	672 (64.9)	812 (78.5)	<0.001	697 (67.3)	787 (76.1)	<0.001
Invasive ventilation, *n* (%)	2069 (99.7)	1938 (93.3)	976 (94.0)	961 (92.6)	0.186	987 (95.1)	951 (91.6)	0.002
RRT, *n* (%)	2069 (99.7)	218 (10.5)	102 (9.9)	116 (11.2)	0.317	116 (11.2)	102 (9.9)	0.352
**Biomarkers**								
IL-6, pg/ml [IQR]	2076 (100)	100.9 [43.5, 261.7]	43.5 [23.8, 69.8]	261.8 [156.4, 604]	<0.001	57.5 [28.2, 129.2]	179.0 [86.3, 417.0]	<0.001
CRP, mg/L [IQR]	2076 (100)	143.7 [78.6, 219.8]	101.2 [52.9, 160.2]	191.8 [127.0, 261.4]	<0.001	78.6 [45.8, 111.4]	219.9 [180.0, 281.9]	<0.001
**Outcome**								
ICU LOS, days [IQR]	2076 (100)	13 [7, 21]	12 [7,21]	13 [8, 22]	0.019	12 [7, 21]	13 [7, 22]	0.038
In-ICU mortality, *n* (%)	2076 (100)	450 (21.7)	151 (14.5)	299 (28.8)	<0.001	205 (19.7)	245 (23.6)	0.038
Day-90 mortality, *n* (%)	2069 (99.7)	627 (30.3)	234 (22.6)	393 (38.0)	<0.001	294 (28.4)	333 (32.2)	0.062

*The FROG-ICU study protocol has been designed before the publications of the sepsis-3 criteria and patients admitted for severe infections have been initially classified into the severe sepsis or septic shock category and were reclassified into the sepsis or septic shock category for the present analysis. High IL-6 or CRP are defined as over the median value. IQR, Interquartile range; SAPS-II, Simplified Acute Physiology Score II; SOFA, Sequential Organ Failure Assessment; GCS, Glasgow Coma Scale; SAP, Systolic arterial pressure; DAP, Diastolic arterial pressure; WBC, White blood cells; PT, Prothrombin time; RRT, Renal replacement therapy; LOS, Length of stay.

### IL-6 and CRP Concentrations

The median IL-6 and CRP concentrations at baseline in the whole population were 100.9 pg/ml [43.5–261.7] and 143.7 mg/L [78.6–219.8], respectively. Concentrations of the two biomarkers were positively correlated with each other (*r*_s_ = 0.49 [0.46–0.52], *p* < 0.001). IL-6 and CRP were not and weakly correlated with white blood cell count: *r*_s_ = −0.0003 [−0.0442–0.0450], *p* = 0.99 and *r*_s_ = 0.098 [0.053–0.142], *p* < 0.001, respectively. The median IL-6 or CRP concentrations at baseline differed according to the main admission diagnosis (**Supplemental Figure S1**). Patients admitted for hemorrhagic shock had the highest concentrations of IL-6 while patients admitted after planned surgery had the highest concentrations of CRP. Admission for a neurological disorder was associated with the lowest concentrations of both biomarkers.

### Characteristics of Concordant and Discordant Groups

To compare the clinical utility of IL-6 and CRP, we further evaluated the impact of biomarker concentrations in concordant (IL-6 and CRP both above or below median value) and discordant groups. Patients were classified into four categories depending on concentrations of both biomarkers relative to median values (CRP^LOW^/IL-6^LOW^, CRP^LOW^/IL-6^HIGH^, CRP^HIGH^/IL-6^LOW^, and CRP^HIGH^/IL-6^HIGH^). The characteristics of these four subgroups are presented in **Supplemental Table S1**. As previously observed, high IL-6 associates with reduced systolic arterial pressure and diastolic arterial pressure, increased serum creatinine and reduced urine output, and higher total bilirubin and lower platelets count. Sub-stratification of clinical and biological variables according to the concentration of CRP highlighted significant and consistent associations only for heart rate (higher in CRP^HIGH^ subgroups) and white blood cell count (higher in CRP^HIGH^ subgroups).

### Association Between IL-6 or CRP Concentrations and Survival

**Figure 1:**
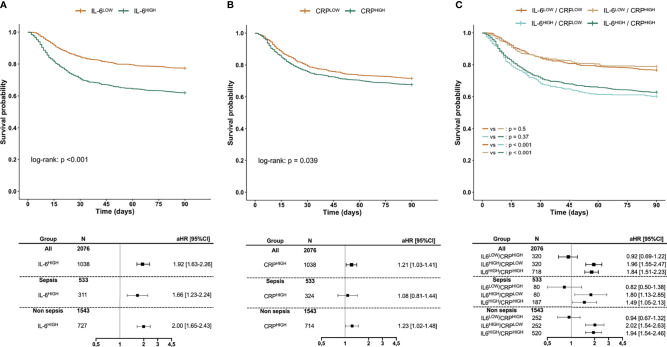
Survival according to IL-6 **(A)**, CRP **(B)**, or both **(C)**. Kaplan–Meier survival curves according to the concentration of IL-6 **(A)**, CRP **(B)**, as well as in concordant or discordant subgroups **(C)** along with aHR estimated using Cox proportional hazard models. IL-6^HIGH^ and CRP^HIGH^ are defined as patients with biomarker over the respective median value. aHRs of day-90 mortality are given for the IL-6^HIGH^ group taking the IL-6^LOW^ group as the reference risk **(A)**, for the CRP^HIGH^ group taking the CRP^LOW^ group as the reference risk **(B)**, and for IL-6^LOW^/CRP^HIGH^, IL-6^HIGH^/CRP^LOW^, and IL-6^HIGH^/CRP^HIGH^ groups taking IL-6^LOW^/CRP^LOW^ as the reference risk **(C)**, after adjustment for SAPS-II. aHR, adjusted Hazard ratio; CI, Confidence interval; SAPS-II, Simplified Acute Physiology Score II.

In the whole cohort, day-90 mortality rate was 30%. The day-90 mortality rate was significantly higher for patients having an IL-6 concentration at baseline higher than median than for those under (38% vs. 23%, *p* < 0.001). Survival could be significantly stratified according to IL-6 concentration at baseline with an unadjusted HR for death of 1.91 [1.62–2.24] for having an IL-6 over 100.9 pg/ml. This association remained significant after adjustment on SAPS-II (aHR 1.92 [1.63–2.26]) and persisted across several subgroups of patients dichotomized according to sepsis or non-septic diagnosis, severity, age, or gender (**Figure 1**; **Supplemental Figure S2**). Conversely, day-90 mortality rate was not significantly different for patients having a baseline supra-median CRP as compared to others (32% vs. 28%, *p* = 0.062). Although survival could be significantly stratified according to CRP concentration at baseline (unadjusted HR 1.18 [1.01–1.38], aHR 1.21 [1.03–1.41]), the association was inconstant across patients’ subgroups (**Figure 1**; **Supplemental Figure S2**). Additionally, for one category of IL-6, taking into account CRP concentration did not refine the survival analysis: Taking IL-6^LOW^/CRP^LOW^ as the reference risk, a statistically significant aHR was only found for sub-groups with high IL-6 (**Figure 1**; **Supplemental Figure S2**).

**Figure 2:**
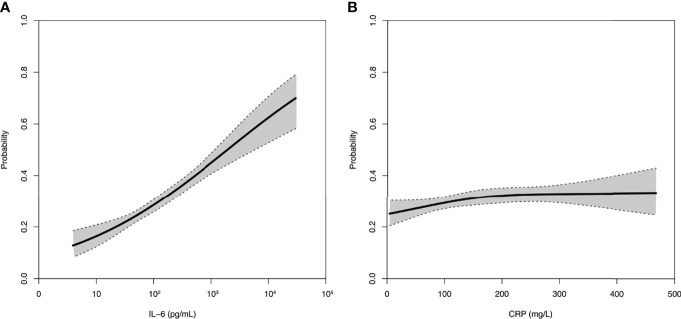
Relation between IL-6 **(A)** or CRP **(B)** and day-90 mortality. Spline curves of the probability of day-90 mortality and associated 95% confidence interval according to the baseline concentration of IL-6 or CRP.

Furthermore, the association between day-90 mortality and IL-6 appeared log-linear with an incremental risk translating into an OR for death of 2.04 [1.76–2.37] per incremental log_10_ IL-6, i.e., for a 10-fold increase. In contrast, the relation between CRP value and day-90 mortality was weak with an OR for death of 1.10 [1.05–1.20] per 100 mg/L increase and tended to reach a plateau (**Figure 2**).

### Association Between IL-6 or CRP Concentration With Organ Dysfunction and Need for Organ Support

**Figure 3:**
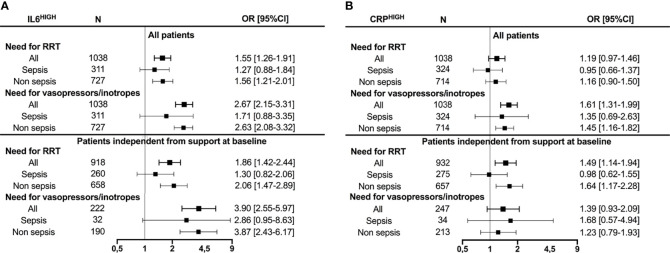
Associations between IL-6 **(A)** or CRP **(B)** and the need for organ support. IL-6^HIGH^ and CRP^HIGH^ are defined as patients with biomarker over the respective median value. ORs for the need for RRT or vasopressors/inotropes are given for the IL-6^HIGH^ group taking the IL-6^LOW^ group as the reference risk **(A)**, for the CRP^HIGH^ group taking the CRP^LOW^ group as the reference risk **(B)**, in the whole cohort, as well as in patients independent from the given support at baseline. OR, Odds ratio; CI, Confidence interval; RRT, Renal replacement therapy.

Baseline IL-6 concentration moderately correlated with SOFA score at inclusion (*r*_s_ = 0.35 [0.3–0.39], *p* < 0.001), and SOFA score gradually increased across IL-6 quartiles (**Supplemental Figure S3**). High IL-6 was associated with the need for organ-support therapies, such as vasopressors/inotropes during ICU stay (OR 2.67 [2.15–3.31]) and for subsequent requirement of vasopressors/inotropes among patients without such support at baseline (OR 3.90 [2.55-5.07]). High IL-6 was associated with the need for RRT during ICU stay (OR 1.55 [1.26–1.91]), and for subsequent requirement of RRT among patients without need for RRT at baseline (OR 1.86 [1.42–2.44]) (**Figure 3**). Conversely, correlation between CRP concentration and SOFA score was low (*r*_s_ = 0.14 [0.09–0.19], *p* < 0.001) with a substantial overlap of SOFA score across CRP quartiles (**Supplemental Figure S3**). High CRP was associated with the need for vasopressors/inotropes (OR 1.61 [1.31–1.99]) during ICU stay, but the association was no longer significant when considering only patients independent from such therapies at baseline (OR 1.39 [0.93–2.09]). Additionally, high CRP was associated with the need for RRT only in patients independent from RRT at baseline (OR 1.49 [1.14–1.94]). Subgroup analysis suggested that associations with the need for organ support were driven by the non-septic subpopulation (**Figure 3**).

### Reclassification Analysis

Using severity scores related to outcome such as SAPS-II and SOFA, we used reclassification analysis to study the ability of each biomarker to improve prognostication. Overall, baseline IL-6 and CRP were associated with an improvement of the mortality risk prediction by SAPS-II while only IL-6 was associated with an improvement of risk prediction evaluated with the SOFA score. In detail, only IL-6 significantly improved the classification of non-survivors in addition to SAPS-II, with a net proportion of non-survivors assigned a higher risk of 10.7% [2.9–18.5]. Both CRP and IL-6 improved the classification of survivors in addition to SAPS-II or SOFA. However, NRI_nonevents_ was consistently higher for IL-6 compared to CRP. Taking into account the two biomarkers resulted in superimposable prediction ability than IL-6 alone (**Table 2**). Thus, considering IL-6, CRP, or both in addition to SAPS-II led to a better classification of 24.2% [19.2–29.2], 16.7% [11.6–21.8], and 27.4% [22.4–32.4] of survivors, respectively. Considering IL-6, CRP, or both in addition to SOFA score led to a better classification of 26.8% [20.9–32.6], 17.1% [11.1–23.0], and 27.3% [21.5–33.1] of survivors, respectively.

**Table 2:** Reclassification analysis.

SAPS-II as the baseline risk			
	IL-6	CRP	IL-6 and CRP
**AUC of ROC curve** [**95% CI**]			
AUC Biomarker	0.625 [0.597–0.65]	0.531 [0.504–0.558]	0.631 [0.603–0.656]
AUC Biomarker + SAPS-II	0.686 [0.661–0.712]*	0.654 [0.629–0.679]	0.689 [0.664–0.712]*
**NRI of biomarker(s) + SAPS-II**			
% Events to higher risk	55.3	46.9	54.2
% Nonevents to higher risk	37.9	41.6	36.3
% Events to lower risk	44.7	53.1	45.8
% Nonevents to lower risk	62.1	58.4	63.7
NRI_events_ [95% CI]	0.107 [0.029–0.185]	−0.062 [-0.14–0.016]	0.085 [0.007–0.163]
NRI_nonevents_ [95% CI]	0.242 [0.192–0.292]	0.167 [0.116–0.218]	0.274 [0.224–0.324]
Total cNRI [95% CI]	0.349 [0.256–0.442]	0.105 [0.012–0.198]	0.359 [0.266–0.451]
**IDI of biomarker(s) + SAPS-II**			
Events to higher risk	0.027	0.002	0.0281
Nonevents to lower risk	0.012	0.001	0.0122
Total (95% CI)	0.038 [0.029–0.048]	0.002 [0–0.005]	0.04 [0.031–0.05]
**SOFA score as the baseline risk**			
	**IL-6**	**CRP**	**IL-6 and CRP**
**AUC of ROC curve** [**95% CI**]			
AUC Biomarker	0.625 [0.598–0.65]	0.531 [0.504–0.558]	0.631 [0.603–0.656]
AUC Biomarker + SOFA	0.64 [0.61–0.674]*	0.588 [0.555–0.621]	0.644 [0.613–0.675]*
**NRI of biomarker(s) + SOFA**			
% Events to higher risk	53.7	46.6	54.1
% Nonevents to higher risk	36.3	41.5	36.3
% Events to lower risk	46.3	53.4	45.9
% Nonevents to lower risk	63.4	58.5	63.7
NRI_events_ [95% CI]	0.073 [−0.019–0.165]	−0.069 [−0.161–0.023]	0.082 [−0.01–0.174]
NRI_nonevents_ [95% CI]	0.268 [0.209–0.326]	0.171 [0.111–0.23]	0.273 [0.215–0.331]
Total cNRI [95% CI]	0.341 [0.232–0.45]	0.102 [−0.008–0.212]	0.355 [0.247–0.464]
**IDI of biomarker(s) + SOFA**			
Events to higher risk	0.028	0.002	0.03
Nonevents to lower risk	0.012	0.001	0.013
Total (95% CI)	0.041 [0.029–0.052]	0.002 [0–0.005]	0.043 [0.031–0.054]

*Indicates a p-value < 0.05 when comparing ROC SAPS-II/SOFA vs. ROC SAPS-II/SOFA + the biomarker with the Delong test. AUC, Area under curve; ROC, Receiver operating characteristic; NRI, Net reclassification index; IDI, Integrative discrimination improvement; CI, Confidence interval.

However, total NRI was consistently higher for IL-6 compared to CRP. Taking into account the two biomarkers resulted in superimposable prediction ability than IL-6 alone (**Table 2**). Thus, considering IL-6, CRP, or both in addition to SAPS-II led to a better classification of 34.9% [25.6–44.2], 10.5% [1.2–19.8], and 35.9% [26.6–45.1] of patients, respectively. Considering IL-6, CRP, or both in addition to SOFA score led to a better classification of 34.1% [23.2–45], 10.2% [−0.8–21.2], and 35.5% [24.7–46.4] of patients, respectively.

## Discussion

Our study shows that in a general population of critically ill patients, high IL-6 and CRP concentrations associate with organ dysfunction, need for organ-support therapies, and worse 90-day survival. However, IL-6 seems to outperform CRP for patient prognostication. In addition, high IL-6, regardless of CRP value, associates with hemodynamic dysfunction characterized by reduced systolic and diastolic arterial pressure, coagulopathy, and hepatic and renal dysfunction. Finally, in reclassification analysis, IL-6 is associated with the greatest improvement of the prediction ability of SAPS-II or SOFA. Altogether, our results suggest that IL-6 should be preferred over CRP for the evaluation of critically patients’ outcomes.

Our study has several strengths. To date, this is the largest study to investigate the prognosis value of IL-6 in critically ill patients. Additionally, as our cohort is composed of a broad range of ICU admission diagnoses, our findings are not limited to septic patients and could be relevant to all critically ill patients. Lastly, few studies compared the prognostic value of IL-6 and CRP. In this regard, we showed a clear advantage of IL-6 over CRP.

Our conclusions join previous work that explored the prognostic interest of IL-6 in critically ill septic patients and confirm that IL-6 measurement is also interesting in non-septic patients. On a physiological perspective, it is noticeable that high IL-6 concentration associates with reduced diastolic arterial pressure, suggesting vasoplegia. This observation is of particular impact in the sub-group of non-septic patients and joins previous observations (9). Thus, vasoplegia, coagulopathy, and renal and hepatic dysfunctions are all mechanisms that could explain why the magnitude of the non-septic systemic inflammatory reaction as evaluated by IL-6 is associated with worse prognosis in these patients. This finding strengthens the interest of IL-6 measurement in this population. Further, very few studies have compared the prognostic impact of measuring IL-6 and CRP. In this perspective, our work strongly suggests that measuring IL-6 is preferable. Additionally, the combined analysis of the two biomarkers seems to show that the addition of CRP provides little benefit as compared to IL-6 alone. Interestingly, the impact of IL-6 on survival seems to be proportional to IL-6 concentration as demonstrated by a log-linear relationship, while the relationship between CRP and mortality seems to rapidly reach a plateau. This result illustrates that elevated CRP is associated with a worse outcome, but the magnitude of elevation is poorly informative.

It may seem surprising that CRP and IL-6 do not provide similar prognostic information given that IL-6 is the main inductor of CRP synthesis. Several factors may account for such discrepancy, including the participation of IL-1β and TNFα to CRP induction (18, 19), the role of liver function (20, 21), polymorphisms at the IL-6 receptor or CRP *locus* (22, 23), as well as the different kinetics of these two biomarkers. Additionally, CRP has been shown to exert regulatory activities such as the counteraction of circulating histone toxicity. Circulating histones are well-known damage-associated molecular patterns associated with IL-6 release, especially in the critical illness context (24, 25). Therefore, it can be hypothesized that the extent of IL-6 release might be directly related to the initial insult, while CRP elevation for a given IL-6 stimulus could also reflect a protective response. More generally, our work meets the fields of clinical immunology and oncology in the assumption that disease activity is best reflected by IL-6 rather than CRP (1).

Our work also has several limitations. We used data and biological samples from a previous study with different objectives. The biological samples were expected to be taken within 24 h after ICU admission. Thus, concentrations obtained after a few hours of delay may be lower than those obtained immediately after admission considering the short half-life of IL-6; this may have influenced our results. Concentrations were consequently higher than the basal level. It is interesting to note, from a real-world perspective, that even a few hours of delay retains prognostic interest.

Additionally, this instantaneous capture of the dynamic course of different conditions is susceptible to influence the respective prognostic value of each biomarker. Thus, for some conditions (e.g., sepsis), the time between disease onset and hospital admission can be substantial. In such situation, peak IL-6 has often already passed while CRP is still rising. In other situations where the delay from disease onset is short, admission captures biomarkers at a time when IL-6 peaks and CRP is just growing. This is susceptible to introducing some disease-specific heterogeneities in the predictive value of the biomarkers. As an example, the lack of performance of IL-6 to predict the need for organ support in the septic population could reflect these differences in time from disease onset.

Furthermore, the main admission category was identified by the local FROG-ICU investigator as the main reason leading to ICU admission according to a predefined list. Therefore, patients with organ dysfunction(s) primarily caused by infection were classified into the sepsis/septic shock category, but it cannot be formally excluded that some patients classified into other admission categories could have presented, in addition to their non-septic primary disease, a mild infection influencing IL-6 and/or CRP concentration and thus, the results of our analysis.

Finally, CRP or IL-6 only reflects one limited aspect (essentially pro-inflammatory) of the host immune response to aggression. As the clinical situation likely results from the complex balance between several immune factors, it is likely that it would be even more interesting to focus on a combination of pro- and anti-inflammatory biomarkers to assess prognosis (8). Nevertheless, as other immune markers are rarely available in a routine fashion, such strategy might be more difficult to implement in clinical practice.

Future work should focus on the best timing and cutoff of IL-6 to assess the prognosis of critically ill patients. The usefulness of monitoring systemic inflammation with serial IL-6 measurement throughout ICU and hospital stay deserves further studies. Additionally, the potential interest of associating the value of IL-6 with clinical prognostic scores at the bedside to determine the prognosis and possibly the patient’s orientation, as suggested by our reclassification analyses, should be considered. Lastly, our work suggests the potential role of IL-6, rather than CRP, as an enrichment biomarker for therapeutic trials of interventions aimed at taming inflammation.

## Conclusion

In a large prospective cohort of critically ill patients including both septic and non-septic patients, we showed that IL-6, rather than CRP, associates with organ dysfunction, need for organ support, and worse 90-day survival. Overall, IL-6 should be preferred over CRP to evaluate critically ill patients’ prognoses and possibly to guide potential therapeutic interventions aimed at taming inflammation.

## Investigators for the FROG-ICU Study

N Deye, C Fauvaux, A Mebazaa, C Damoisel, D Payen, Hopital Lariboisiere (Paris); E Azoulay, AS Moreau, L Jacob, O Marie, Hopital Saint Louis (Paris); M Wolf, R Sonneville, R Bronchard, Hopital Bichat (Paris); I Rennuit, C Paugam, Hopital Beaujon (Clichy); JP Mira, A Cariou, A Tesnieres, Hopital Cochin (Paris); N Dufour, N Anguel, L Guerin, J Duranteau, C Ract, Hopital Bicetre (Le Kremlin-Bicetre); M Leone, B Pastene, Chu De Marseille (Marseille); T Sharshar, A Fayssoyl, Hopital Raymond Poincare (Garches); J-L Baudel, B Guidet, Hopital Saint-Antoine; Q Lu, W Jie Gu, N Brechot, A Combes, Hopital De La Pitie—Salpetriere (Paris); S Jaber, A Pradel, Y Coisel, M Conseil, Chu St Eloi (Montpellier); A Veillard Baron, L Bodson, Hopital Ambroise Pare (Boulogne); Jy Lefrant, L Elotmani, A Ayral, S Lloret, Chu Caremeau (Nimes); S Pily-Flouri, Jb Pretalli, Hopital Jean Minjoz (Besançon); Pf Laterre, V Montiel, Mf Dujardin, C Berghe, Clinique Saint-Luc (Belgium)

## Data Availability Statement

All data analyzed in this report are available upon reasonable request to the corresponding author. Requests to access these datasets should be directed to etienne.gayat@aphp.fr.

## Ethics Statement

The studies involving human participants were reviewed and approved by Comité de Protection des Personnes—Ile de France IV, IRB n°00003835 Commission d’éthique biomédicale hospitalo-facultaire de l’hôpital de Louvain, IRB n°B403201213352. Written informed consent for participation was not required for this study in accordance with the national legislation and the institutional requirements.

## Author Contributions

All the authors listed meet the authorship criteria. AP, LM, AM, and BC designed the study and contributed to data analysis and interpretation and drafting of the manuscript. CR, MS, EG, BD, and GC critically reviewed the analysis and made substantial contribution to the manuscript. All authors reviewed the manuscript and approved the final version.

## Funding

This study received funding from the Programme Hospitalier de la Recherche Clinique (AON 10-216) and by a research grant from the Société Française d’Anesthésie—Réanimation. Abbott, Sphingotec, Roche Diagnostics, and Critical Diagnostics provided unrestricted free kits to Assistance Publique—Hôpitaux de Paris to conduct biomarker analyses. The funders were not involved in the study design, collection, analysis, interpretation of data, the writing of this article, or the decision to submit it for publication

## Conflict of Interest

BC was a member of an advisory board for Roche diagnostic. BD and GC are employees of Momentum Research and who received research grants from Abbott Laboratories, Amgen, Celyad, Cirius Therapeutics, Corteria Pharmaceuticals, Roche Diagnostics, Sanofi, Sulfagenix, Windtree Therapeutics Inc., and XyloCor Therapeutics Inc. CR received a research grant from Zoll Foundation. AM received speaker’s honoraria from Abbott, Novartis, Orion, Roche, and Servier, and fees as a member of the advisory board and/or steering committee from Cardiorentis, Adrenomed, MyCartis, Neurotronik, and Sphingotec. EG received a research grant from Sphingotec, and consultancy fees from Magnisense and Roche Diagnostics.

The remaining authors declare that the research was conducted in the absence of any commercial or financial relationships that could be construed as a potential conflict of interest.

## Publisher’s Note

All claims expressed in this article are solely those of the authors and do not necessarily represent those of their affiliated organizations, or those of the publisher, the editors and the reviewers. Any product that may be evaluated in this article, or claim that may be made by its manufacturer, is not guaranteed or endorsed by the publisher.

## Abbreviations

IL-6, Interleukin-6; CRP, C-reactive protein; ICU, Intensive care unit; FROG-ICU, French and euRopean Outcome reGistry in Intensive Care Units; SAPS-II, Simplified Acute Physiology Score II; SOFA, Sequential Organ Failure Assessment; (a)HR, (adjusted) Hazard ratio; OR, Odds ratio; CI, Confidence interval; NRI, Net reclassification index; IDI, Integrative discrimination improvement; RRT, Renal replacement therapy.
