# Supplementary figures and images for: Systemic Inflammation Evaluated by Interleukin-6 or C-Reactive Protein in Critically Ill Patients: Results From the FROG-ICU Study
Source: Front Immunol. 2022 May 12;13:868348. doi: 10.3389/fimmu.2022.868348 (PMC9134087; doi:10.3389/fimmu.2022.868348)

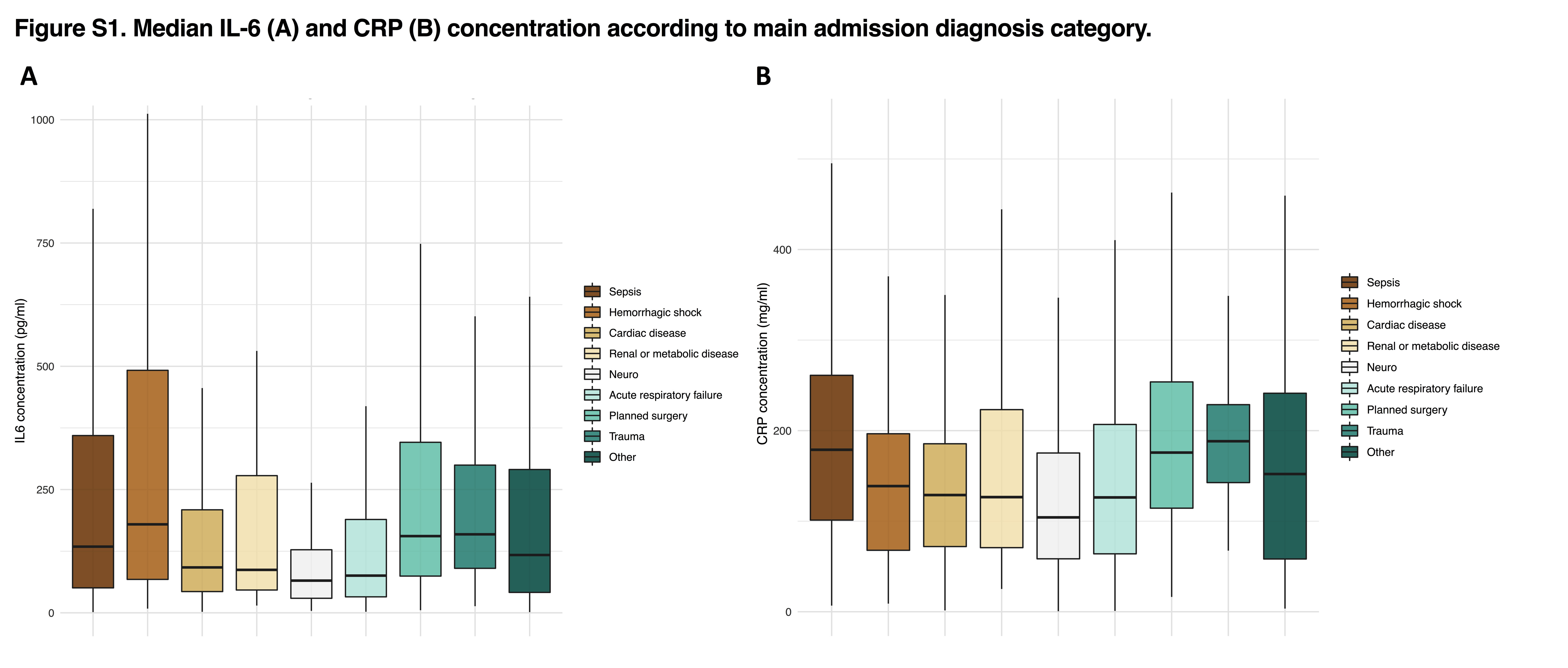

Supplement: Supplementary file 1 [file Image_1.tiff]

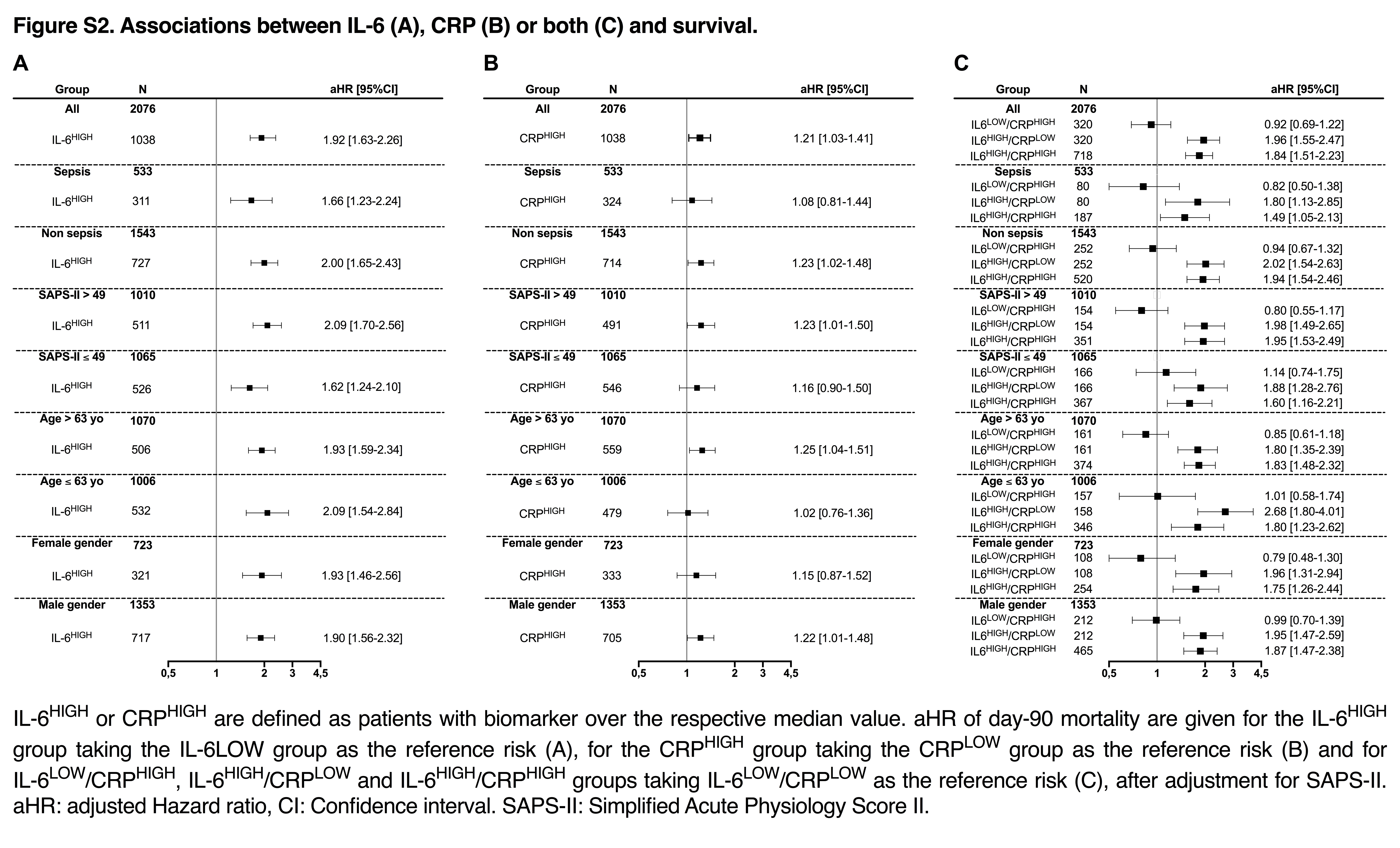

Supplement: Supplementary file 2 [file Image_2.tiff]

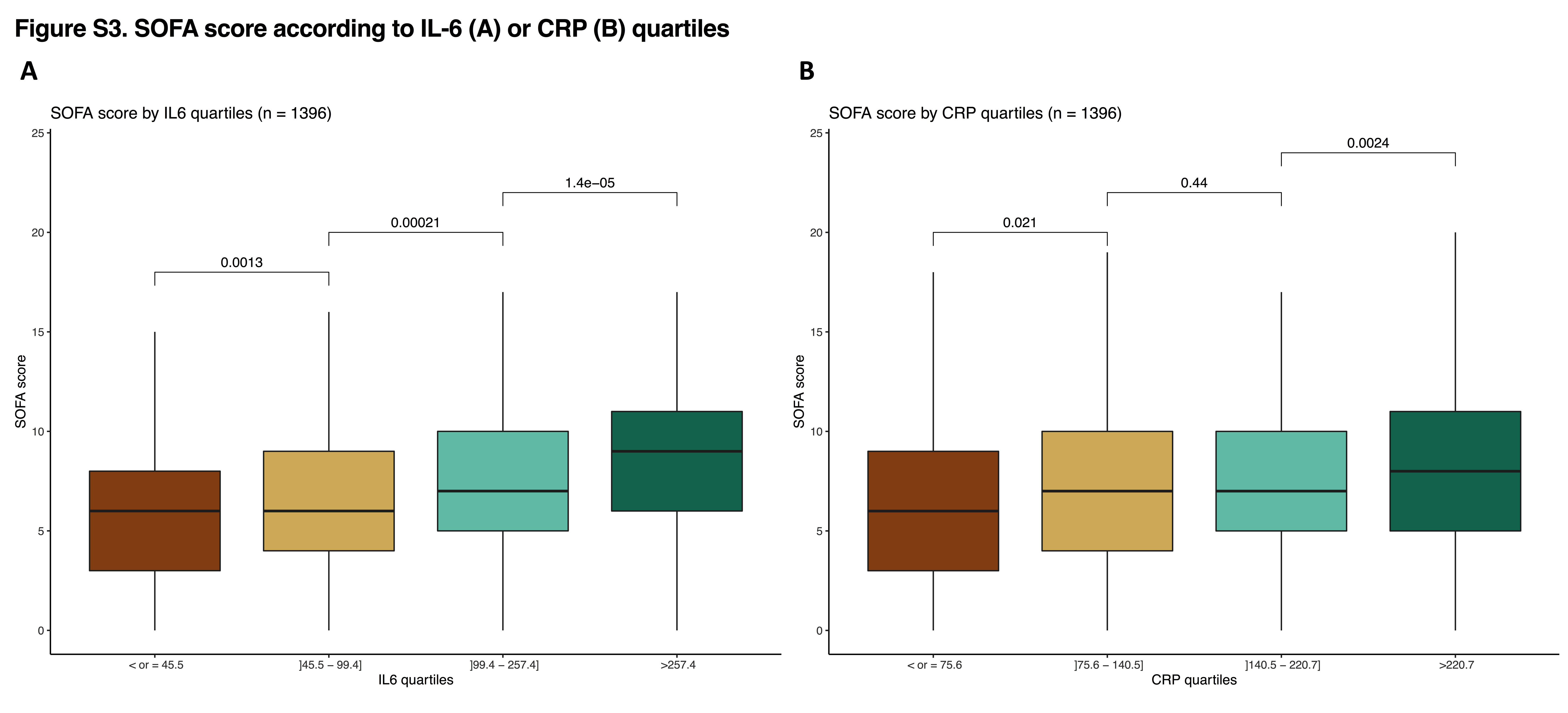

Supplement: Supplementary file 3 [file Image_3.tiff]
