# Supplementary material for: Systemic Inflammation Evaluated by Interleukin-6 or C-Reactive Protein in Critically Ill Patients: Results From the FROG-ICU Study
Source: Front Immunol. 2022 May 12;13:868348. doi: 10.3389/fimmu.2022.868348 (PMC9134087; doi:10.3389/fimmu.2022.868348)
Supplement: Supplementary file 4 [file Table_1.docx]

**Table S1. Patients‘ characteristics according to the concentrations of both biomarkers**

|  | **Available data**  N (%) | **IL-6^LOW^/CRP^LOW^**  **N=718** | **IL-6^LOW^/CRP^HIGH^**  **N=320** | **p** | **IL-6^HIGH^/CRP^LOW^**  **N=320** | **IL-6^HIGH^/CRP^HIGH^**  **N=718** | **p** |
| --- | --- | --- | --- | --- | --- | --- | --- |
| **Patients characteristics** |  |  |  |  |  |  |  |
| Age, years [IQR] | 2076 (100) | 63 [49,73] | 64 [51,74] | 0.290 | 61 [50,73] | 64 [53,75] | 0.016 |
| Gender, female n (%) | 2076 (100) | 290 (40.4) | 112 (35.0) | 0.112 | 100 (31.2) | 221 (30.8) | 0.885 |
| SAPS-II, points [IQR] | 2075 (99.9) | 49 [36,62] | 47 [33,62] | 0.236 | 50 [38,64] | 49 [36,62] | 0.416 |
| SOFA, points [IQR] | 1510 (72.7) | 7 [4,10] | 7 [4,10] | 0.527 | 8 [6,11] | 8 [5,10] | 0.069 |
| **Comorbidities** |  |  |  |  |  |  |  |
| Charlson comorbidity index | 2076 (100) | 1 [0,3] | 1 [0, 2] | 0.376 | 2 [0,3] | 1 [0,2] | 0.027 |
| Hypertension n (%) | 2072 (99.8) | 298 (41.6) | 143 (44.7) | 0.377 | 118 (36.9) | 339 (47.3) | 0.002 |
| Diabetes mellitus, n (%) | 2072 (99.8) | 142 (19.8) | 68 (21.2) | 0.616 | 43 (13.4) | 130 (18.2) | 0.071 |
| Chronic heart failure, n (%) | 2072 (99.8) | 53 (7.4) | 22 (6.9) | 0.797 | 26 (8.1) | 51 (7.1) | 0.608 |
| Chronic kidney disease, n (%) | 2072 (99.8) | 69 (9.6) | 34 (10.6) | 0.653 | 36 (11.2) | 101 (14.1) | 0.234 |
| Chronic liver disease, n (%) | 2072 (99.8) | 59 (8.2) | 6 (1.9) | <0.001 | 61 (19.1) | 32 (4.5) | <0.001 |
| Cancer, n (%) | 2072 (99.8) | 73 (10.2) | 34 (10.6) | 0.826 | 49 (15.3) | 123 (17.2) | 0.471 |
| **Admission category** | 2073 (99.9) |  |  |  |  |  |  |
| Sepsis and septic shock, n (%) |  | 147 (20.5) | 75 (23.4) | 0.287 | 62 (19.4) | 249 (34.7) | <0.001 |
| Hemorrhagic shock, n (%) |  | 23 (3.2) | 13 (4.1) | 0.468 | 38 (11.9) | 36 (5.0) | <0.001 |
| Cardiac arrest or cardiogenic shock, n (%) |  | 129 (18.0) | 47 (14.7) | 0.21 | 59 (18.4) | 87 (12.1) | 0.009 |
| Renal and metabolic disease, n (%) |  | 13 (1.8) | 5 (1.6) | 1.000 | 7 (2.2) | 8 (1.1) | 0.257 |
| Neurological disorder, n (%) |  | 147 (20.5) | 46 (14.4) | 0.020 | 39 (12.2) | 52 (7.2) | 0.012 |
| Acute respiratory failure, n (%) |  | 173 (24.1) | 66 (20.6) | 0.232 | 44 (13.8) | 109 (15.2) | 0.571 |
| Planned surgery, n (%) |  | 34 (4.7) | 21 (6.6) | 0.232 | 27 (8.4) | 83 (11.6) | 0.155 |
| Trauma, n (%) |  | 8 (1.1) | 19 (5.9) | <0.001 | 16 (5.0) | 46 (6.4) | 0.478 |
| Others, n (%) |  | 44 (6.1) | 28 (8.8) | 0.145 | 28 (8.8) | 48 (6.7) | 0.247 |
| **Clinical and biological data at admission** |  |  |  |  |  |  |  |
| GCS, points [IQR] | 1301(62.7) | 13 [5,15] | 13 [3,15] | 0.521 | 9 [3,15] | 11 [3,15] | 0.159 |
| PaO_2_/FiO_2_, mmHg [IQR] | 1776 (85.5) | 268 [194,367] | 256 [182,341] | 0.083 | 253 [188, 343] | 223 [158,304] | 0.001 |
| Heart rate, beats/min [IQR] | 1999 (96.3) | 87 [74,101] | 90 [77,106] | 0.006 | 91 [80,107] | 96 [83,110] | 0.041 |
| SAP, mmHg [IQR] | 2027 (97.6) | 128 [112,147] | 124 [110, 140] | 0.008 | 116 [104, 134] | 118 [105, 134] | 0.555 |
| DAP, mmHg [IQR] | 1957 (94.3) | 65 [55,74] | 62 [55,71] | 0.047 | 58 [52,67] | 58 [51,67] | 0.524 |
| MAP, mmHg [IQR] | 1957 (94.3) | 86 [76,97] | 83 [75,92] | 0.005 | 78 [70,88] | 78 [71,88] | 0.951 |
| Hemoglobin, g/dL [IQR] | 1973(95.0) | 10.2 [9.1,11.6] | 10.1 [8.9,11.7] | 0.938 | 9.8 [8.9,11.5] | 9.8 [8.9,11.2] | 0.291 |
| WBC, 10^9^/L [IQR] | 1933 (93.1) | 10.3 [7.5,15.3] | 11.7 [8.2,16.6] | 0.018 | 10.1 [6.7,15.5] | 11.5 [7.7,17.0] | 0.030 |
| Urine output, mL [IQR] | 1711 (82.4) | 1500 [900,2400] | 1350 [800,2100] | 0.013 | 1200 [684,1988] | 1300 [700,1950] | 0.895 |
| Creatinine, µmol/L [IQR] | 1987 (95.7) | 73 [53,121] | 82 [60,147] | 0.006 | 94 [61,160] | 100 [66,171] | 0.179 |
| Lactate, µmol/L [IQR] | 1638 (78.9) | 1.3 [0.9,1.7] | 1.3 [1.0,1.8] | 0.824 | 1.5 [1.1,2.2] | 1.5 [1.1,2.2] | 0.596 |
| Bilirubin, µmol/L [IQR] | 1293 (62.3) | 10 [7,19] | 12 [8,22] | 0.172 | 22 [10,64] | 14 [9,27] | <0.001 |
| PT rate, % [IQR] | 1147 (55.3) | 73 [63,84] | 72 [62,83] | 0.843 | 61 [43,75] | 65 [53,76] | 0.011 |
| Platelets, 10^9^/L [IQR] | 1950 (93.9) | 189 [122,266] | 184 [115,261] | 0.364 | 126 [78,196] | 147 [91,228] | 0.010 |
| **Organ support at baseline** |  |  |  |  |  |  |  |
| Vasopressors, n (%) | 2069 (99.7) | 464 (64.9) | 208 (65.0) | 1.000 | 233 (72.8) | 579 (81.1) | 0.003 |
| Invasive ventilation, n (%) | 2069 (99.7) | 680 (94.7) | 297 (92.8) | 0.253 | 307 (95.9) | 654 (91.1) | 0.005 |
| RRT, n (%) | 2069 (99.7) | 76 (10.6) | 26 (8.1) | 0.259 | 40 (12.5) | 76 (10.6) | 0.395 |
| **Biomarkers** |  |  |  |  |  |  |  |
| IL-6, pg/mL [IQR] | 2076 (100) | 37.2 [20.1,60.9] | 61.6 [37.0,81.5] | <0.001 | 220.1 [137.4,455.6] | 296.6 [169.2,674.2] | <0.001 |
| CRP, mg/L [IQR] | 2076 (100) | 69.7 [40.0,106.5] | 200.8 [169.0,248.3] | <0.001 | 95.2 [60.8,118.4] | 231.2 [188.2,296.8] | <0.001 |
| **Outcome** |  |  |  |  |  |  |  |
| ICU LOS, days [IQR] | 2076 (100) | 12 [7,21] | 12 [7,20] | 0.613 | 12 [7,21] | 14 [8,24] | 0.023 |
| In-ICU mortality, n (%) | 2076 (100) | 108 (15.0) | 43 (13.4) | 0.567 | 97 (30.3) | 202 (28.1) | 0.504 |
| Day-90 mortality, n (%) | 2069 (99.7) | 167 (23.3) | 67 (21.1) | 0.469 | 127 (39.8) | 266 (37.2) | 0.446 |

IL-6^HIGH^ or CRP^HIGH^ are defined as patients with biomarker over the respective median value. IQR: Interquartile range, SAPS-II: Simplified acute Physiology score II, SOFA: Sequential organ failure assessment, GCS: Glasgow coma scale, SAP: Systolic arterial pressure, DAP: Diastolic arterial pressure, MAP: Mean arterial pressure, WBC: White blood cells, PT: Prothrombin time, RRT: Renal replacement therapy, LOS: Length of stay.
